# Associations between depressive symptoms and fronto-temporal activities during a verbal fluency task in patients with schizophrenia

**DOI:** 10.1038/srep30685

**Published:** 2016-07-28

**Authors:** Shenghong Pu, Kazuyuki Nakagome, Akihiko Miura, Masaaki Iwata, Izumi Nagata, Koichi Kaneko

**Affiliations:** 1 Division of Neuropsychiatry, Department of Brain and Neuroscience, Tottori University Faculty of Medicine, 36-1 Nishi-cho, Yonago, Tottori 683-8504, Japan; 2 National Center of Neurology and Psychiatry, 4-1-1 Ogawa-Higashi, Kodaira, Tokyo 187-8551, Japan

## Abstract

Though depressive symptoms are common in patients with schizophrenia, they are often left untreated and are associated with a high relapse rate, suicidal ideation, increased mortality, reduced social adjustment, and poor quality of life. The present study aims to elucidate the association between depressive symptoms and fronto-temporal activities during a cognitive task in patients with schizophrenia. The fronto-temporal activities of 41 Japanese patients with schizophrenia was evaluated during a verbal fluency task using 52-channel near-infrared spectroscopy (NIRS). Depressive symptoms were assessed using the depression/anxiety component of the Positive and Negative Syndrome Scale (PANSS) five-factor model. The depression/anxiety component of the PANSS five-factor model was negatively correlated with activities of the ventrolateral prefrontal cortex (PFC), right dorsolateral PFC, and left temporal regions. Our findings suggest that reduced fronto-temporal activities on NIRS during a verbal fluency task is related to depressive symptom severity in patients with schizophrenia.

The Positive and Negative Syndrome Scale (PANSS) is widely used in the clinical assessment of schizophrenia. Originally, it was divided into three scales: positive, negative, and general psychopathology^1^. However, subsequent factorial analyses indicated the existence of other components. Recently, multiple-factor models of the PANSS have been used to evaluate the multidimensional symptoms of schizophrenia^2^,^3^. Among these, the so-called depressive or anxio-depressive dimension has been frequently used. Evidence collected using this model does not vary from clinical observations. Indeed, depressive symptoms are among the earliest and most frequent signs of schizophrenia onset^4^,^5^, and around 70% of patients with chronic schizophrenia without a major depressive disorder show some signs of depressive symptoms^6^. Depressive symptoms in schizophrenia are known to have detrimental consequences such as a high relapse rate, suicidal ideation, increased mortality, reduced social adjustment, and poor quality of life^4^,^7^,^8^,^9^. Despite the clinical importance of depressive symptoms in patients with schizophrenia, little is known regarding the underlying neurobiological mechanism.

Numerous studies have mostly used functional magnetic resonance imaging (fMRI) and electroencephalography methods to reveal the neural correlates of positive^10^,^11^ or cognitive^12^,^13^ symptoms in schizophrenia. Multichannel near-infrared spectroscopy (NIRS), a functional neuroimaging technology widely used in recent years, can measure hemodynamic changes over the surface of the cortices of the bilateral fronto-temporal regions^14^. This technique enables the detection of spatiotemporal characteristics of brain function by measuring the concentrations of oxygenated haemoglobin (oxy-Hb) and deoxygenated haemoglobin (deoxy-Hb), which are assumed to reflect the regional cerebral blood volume as demonstrated by good correlations with fMRI signals^15^. Numerous studies performed using this technique have consistently shown that, during a verbal fluency task (VFT), oxy-Hb change in the fronto-temporal brain regions is significantly lower in patients with schizophrenia than in healthy controls^16^,^17^,^18^. Further, NIRS measurement during a VFT has been shown to be a useful clinical auxiliary laboratory test for the differential diagnosis of depressive states^9^. Many investigators have reported an association between NIRS signals and clinical symptoms of schizophrenia, suggesting that the fronto-temporal hemodynamic responses during the VFT are related to positive, negative, or general psychopathology symptoms^18^,^19^,^20^. However, these studies did not examine depressive symptoms. Use of specific neurobiological markers (e.g., NIRS) in addition to the five-factor model of PANSS assessment might improve the evaluation of depressive symptoms. Therefore, investigating the five-factor model of PANSS is important for understanding the depressive symptoms of schizophrenia. In this study, we investigated the association between depressive symptoms and fronto-temporal hemodynamic responses during a VFT in patients with schizophrenia using the five-factor model of the PANSS.

According to a review by Drevets^21^, a complex relationship exists between depressive symptom severity and frontal lobe function, i.e., the metabolic activity in the orbital cortex and ventrolateral prefrontal cortex (PFC). Noda *et al*.^22^ recently described an association between VFT-related oxy-Hb change in the right dorsolateral PFC and severity of depressive symptoms in patients with major depressive disorder (MDD). These studies suggest that abnormal functions of the PFC are mood-dependent^22^,^23^,^24^. Based on the preliminary results of previous neuroimaging studies in patients with MDD, we hypothesize that the severity of depressive symptoms would be associated with abnormal PFC hemodynamic responses during a VFT in patients with schizophrenia. Structural brain imaging and neuropsychological studies have provided indirect support of the notion that depressed mood in schizophrenia may have at least partially overlapping neurobiology with major depressive disorder, such as volumetric abnormalities of the frontal and temporal lobes^25^ as well as attentional impairment^26^. The findings of Kohler *et al*.^25^,^26^ offer further support to the notion that depressed mood, regardless of its aetiology, involves similar brain systems, namely the frontal cortex, which is also required for attentional processing. In the present study, considering the consistent finding of attenuated oxy-Hb changes during VFT in the fronto-temporal regions in patients with schizophrenia, we hypothesized that oxy-Hb changes during VFT as measured using NIRS may act as objective indicators of depressive symptom severity.

## Results

### General Demographics

The participants’ demographic data are presented in Table 1.

### Mean Hb changes during the VFT

The mean oxy-Hb levels during the task period were significantly higher than the pre-task baseline for 37 channels (ch2, ch9, ch12, ch13, ch15, ch19 to ch26, ch28 to ch30, and ch32 to 52; 52-channel FDR-corrected *p* < 0.05) in participants with schizophrenia.

The mean deoxy-Hb levels during the task period were significantly higher than those for the pre-task baseline for 28 channels (ch20, ch23 to ch25, ch28 to ch36, and ch38 to 52; 52-channel FDR-corrected *p* < 0.05) in participants with schizophrenia.

### Correlation between the mean Hb changes and clinical symptoms

No significant correlation was found between changes in mean oxy-Hb in any channel and the total, positive, negative, and general psychopathology scores of the PANSS (52-channel FDR-corrected *p* > 0.05).

**Figure 1:**
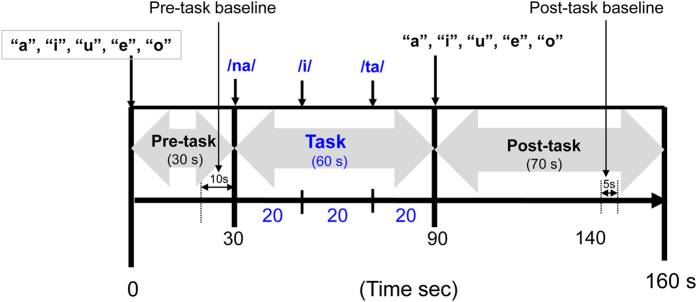
The task design of the verbal fluency task (VFT).

Mean oxy-Hb changes were significantly negatively correlated with depression/anxiety component scores in the ventrolateral PFC (ch23, ch34, and ch50; 52-channel FDR-corrected *p* < 0.05; rho: −0.48 to −0.43), right dorsolateral PFC (ch24, 52-channel FDR-corrected *p* < 0.05; rho = −0.44), and left temporal (ch42 and ch52; 52-channel FDR-corrected *p* < 0.05; rho: −0.47 to −0.46) regions (Fig. 1). However, there was no significant correlation between mean oxy-Hb and scores in any of the other four dimensions (52-channel FDR-corrected *p* > 0.05).

No significant correlation was observed in any channel between changes in mean deoxy-Hb and component five-factor PANSS scores in participants with schizophrenia (52-channel FDR-corrected *p* > 0.05).

All six channels that were significantly correlated with depression/anxiety component scores significantly contributed to depression/anxiety component scores, as revealed by multiple regression analyses (β = −0.517 to −0.340; *p* < 0.05; Table 2). Age, task performance on the VFT, GAF score, duration of illness, and daily dosage of antipsychotic drugs did not significantly contribute to cortical activity. Premorbid IQ and gender significantly contributed to one channel each (ch23 and ch52, respectively; Table 2).

## Discussion

In this study, we measured changes in Hb concentration in the PFC and temporal cortex during a VFT by using 52-channel NIRS imaging. To our knowledge, this is the first study performed using multi-channel NIRS to identify the relationship between VFT-related hemodynamic responses in the fronto-temporal regions and depressive symptoms in patients with schizophrenia. Notably, ventrolateral PFC, right dorsolateral PFC, and left temporal hemodynamic responses during the VFT were correlated with depression/anxiety component scores of the PANSS, even after controlling for VFT performance and demographic factors. These results suggest an association between fronto-temporal activities and depression/anxiety symptom severity in patients with schizophrenia; further, NIRS imaging may be helpful in understanding the neural basis of depressive symptoms.

According to a review by Drevets^21^, a complex relationship exists between depression symptom severity and metabolic activity in the orbital cortex and ventrolateral PFC. Noda *et al*.^22^ recently described an association between VFT-related oxy-Hb change in the right dorsolateral PFC and severity of depressive symptoms in patients with MDD. Even in the absence of direct effects of anxiety on performance, some studies have shown that anxiety may be associated with prefrontal activity^27^,^28^,^29^. A study performed using fMRI observed that lower dorsolateral and ventrolateral PFC activities^27^ corresponded to higher state anxiety, whereas lower dorsolateral and ventral PFC activities corresponded to higher trait anxiety^27^,^30^. A study on generalized anxiety disorder revealed that more severe anxiety symptoms are associated with decreased activities of the ventrolateral PFC in response to emotional stimuli^31^. The findings of these studies indicate that abnormal PFC function is associated with pathological depression/anxiety. In the present study, we observed that oxy-Hb change during VFT was negatively correlated with depression/anxiety component scores of the PANSS in the ventrolateral PFC and right dorsolateral PFC regions. The depression/anxiety component consists of 5 items, i.e., somatic concern (G1), anxiety (G2), feelings of guilt (G3), depression (G6), and preoccupation (G15), and focuses on emotional distress as a unique symptom of schizophrenia^32^. Emotional distress is comprehensively described as the reaction of an individual to external and internal stressors and is characterized by a mixture of sub-threshold distress symptoms such as obsessiveness, depression, hostility, hypersensitivity, anxiety, and paranoid ideation^33^,^34^. Myin Germeys^35^ indicated that increased emotional reactivity is negatively correlated with cognitive impairment in patients with schizophrenia. Data on emotional processes suggest a common neural network involving the PFC, amygdala, insula, basal ganglia, and anterior cingulate^36^,^37^. The PFC, specifically the ventrolateral PFC, is a brain area that is critical for the integration of emotional information and the regulation of the intensity of emotional responses^38^,^39^. Cognitive control processes supported by the dorsolateral PFC have indeed been revealed to modulate the prediction, generation, interpretation, or regulation of emotions and to translate affectively valenced motivational states into goal-oriented behavior^40^,^41^. This general pattern of reduced prefrontal signalling in the context of maintaining affective information is consistent with a body of evidence indicating that schizophrenia is associated with reduced dorsolateral PFC signals during cognitive control and working memory tasks^42^. Ursu *et al*.^41^ suggested that schizophrenia is characterized by a failure of prefrontal circuitry supporting the link between emotion and goal-directed behaviour, arguing that the failure of this mechanism may contribute to deficits in processes related to emotion-cognition interaction. Anticevic and Corlett^43^ argue that this cardinal “emotional” symptom of psychosis can, at least in part, be conceptualized as a breakdown in the interaction of emotion and cognition. Taken together, these findings suggest that the hemodynamic response observed in the PFC region during the VFT in the present study was associated with depressive symptoms. The functional properties of the PFC are particularly important from a clinical point of view, since they may provide insight into to improve depressive symptoms in subjects with schizophrenia.

In the present study, we further observed that oxy-Hb change during VFT was negatively correlated with depression/anxiety component scores in the left superior temporal cortical region. Structural brain imaging and neuropsychological studies have indirectly shown that depressed mood in schizophrenia and MDD may have at least partially overlapping neurobiology, such as volumetric abnormalities of the temporal lobe and anterior cingulate^8^,^44^. A very recent NIRS imaging study observed that oxy-Hb change during VFT in the left temporal regions is associated with depression symptom severity in patients with bipolar disorder^43^. Therefore, fronto-temporal activity as measured using NIRS may be associated with depression/anxiety symptoms, irrespective of diagnosis.

Depressive symptoms are particularly ubiquitous in the disease process or clinical staging of various psychiatric disorders^45^. Depressive symptoms that fulfil the operational diagnostic criteria for a depressive episode/major depression can also occur at any stage of schizophrenia, and depressive symptoms in schizophrenia are known to have detrimental consequences such as a high relapse rate, suicidal ideation, increased mortality, reduced social adjustment, and poor quality of life^4^,^7^,^8^,^9^. Despite the clinical significance of depressive symptoms in patients with schizophrenia, little is known regarding the underlying neurobiological correlates. The results of the present study indicate that fronto-temporal activities measured using NIRS are significantly correlated with depressive symptoms in patients with schizophrenia. Whether the similar finding can be obtained across diagnostic categories is beyond the scope of the study and awaits further studies using patients with various psychiatric diseases.

Interestingly, a correlation was observed only between five-factor model PANSS scores and neural activity in the PFC. Significant relationships were not observed between the original form of PANSS scores (positive, negative, and general psychopathology) and NIRS data. The present finding is consistent with those of previous studies on patients with schizophrenia indicating that the hemodynamic response in the PFC during a cognitive task was related to the five-factor model of the PANSS, but not to the severity of symptoms as evaluated using the conventional PANSS subscales^46^,^47^. Marumo *et al*.^46^ reported an association between disorganization factor scores and less pronounced changes in deoxy-Hb levels in the PFC region during a category fluency task. Further, Nishimura *et al*.^47^ indicated an association between less pronounced changes in oxy-Hb levels in the PFC region during a Go/NoGo task and excitement factor scores as assessed by the five-factor model of the PANSS in patients with schizophrenia. Dysregulation in such distributed neural systems is thought to cause diverse symptoms such as disturbances in perception (hallucinations) and belief (delusions) and emotional dysfunction (amotivation and anhedonia), as well as severe deficits in complex cognitive operations such as working memory, long-term memory, and executive functioning^43^,^48^. Taken together, these findings indicate that NIRS imaging maybe helpful in understanding the neural basis of the five-factor model of the PANSS, and that the five-factor model scores of schizophrenic patients may better reflect PFC dysfunction, which is a plausible neural basis of schizophrenia, than the conventional PANSS subscales used for the evaluation of these patients.

The results of the present study must be interpreted with caution due to certain limitations. First, multi-channel NIRS has limited spatial resolution, unlike fMRI and positron emission tomography. Second, because the analysis was based on cross-sectional data, causality could not be determined. Longitudinal studies are required to assess the cause-and-effect relationships. Third, our sample size was not large and is thus subject to type II errors. Further studies involving larger numbers of patients with schizophrenia are required. Further limitations include lack of reliable information on the proportion of patients who met the DSM criteria for current/past MDD and possible confounding effects of different types and dosages of medications. Future longitudinal investigations should be conducted while monitoring diagnoses of depression and medications. Indeed, medication usage may have influenced the results of the present study. Though only three patients in the study were taking antidepressants, all patients were taking antipsychotics at the time of evaluation, the effects of which should not be ignored. However, we found no significant relationship between chlorpromazine equivalent dosage of daily antipsychotic drugs and depression/anxiety component scores (rho = 0.192; *p* > 0.05). Although antipsychotic dosage levels exhibited significant negative correlations with oxy-Hb changes in two channels (ch10: rho = −0.421, *p* = 0.020; ch13: rho = 0.404, *p* = 0.012), those channels (ch23, ch24, ch34, ch42, ch50, and ch52) in which we observed significant correlation between oxy-Hb changes and depression/anxiety component scores were not among these two channels. Therefore, we suspect that the effect of medication on the results may be minimal.

In conclusion, despite these limitations, our results revealed that fronto-temporal activity measured by NIRS is significantly correlated with the PANSS five-factor model in patients with schizophrenia. Such a result may be particularly relevant for the depression/anxiety component and suggests that assessment and training for fronto-temporal activities might improve outcomes in patients with schizophrenia. Further studies with larger sample sizes are required to verify our findings. If confirmed, our findings may allow investigators to evaluate the potential of NIRS as a neuroimaging biomarker for the assessment of depression/anxiety components in patients with schizophrenia.

## Materials and Methods

### Subjects

Forty-one Japanese patients with schizophrenia who were outpatients at the Tottori University Hospital were included in the study (Table 1). All patients were diagnosed by experienced psychiatrists (A.M., M.I., or I.N.) based on the criteria specified in the fourth edition of the *Diagnostic and Statistical Manual of Mental Disorders* (DSM-IV, American Psychiatric Association 1994)^49^ by using the Mini-International Neuropsychiatric Interview^50^. All patients were receiving treatment with antipsychotics, anxiolytics, and/or antiparkinsonian agents during the study. Fourteen patients were taking olanzapine; 10, aripiprazole; eight, blonanserin; four, quetiapine; three, risperidone; and two, perospirone. The exclusion criteria were as follows: any comorbid neurological illness, previous traumatic brain injury with any known cognitive consequences or loss of consciousness lasting more than 5 min, a history of electroconvulsive therapy, and alcohol/substance abuse or addiction.

All participants provided written informed consent and were recruited from January 2008 to December 2012 on the basis of consecutive referrals. All participants were right-handed according to the Edinburgh Handedness Inventory^51^ and were native Japanese speakers. Premorbid IQ was estimated using the Japanese version of the National Adult Reading Test^52^. The study was approved by the Ethics Committee of the Tottori University Faculty of Medicine (approval No., 885), and the investigation was conducted in accordance with the latest version of the Declaration of Helsinki.

### Clinical evaluation

On the day of the NIRS experiment, psychiatric symptoms and global functioning were evaluated by the same psychiatrist (A.M., M.I., or I.N.) using the PANSS^1^ and the Global Assessment of Functioning scale (GAF; American Psychiatric Association, 1994)^49^, respectively. We used a five-factor model of PANSS as proposed by Lindenmayer *et al*.^32^. The component subscales of this model were as follows: positive (total scores of P1, P5, P6, and G9), negative (total scores of N1, N2, N3, N4, N6, and G16), excitement (total scores of P4, P7, G4, and G14), depression/anxiety (total scores of G1, G2, G3, G6, and G15), and cognitive (total scores of P2, N5, G5, G10, and G11).

### Cognitive task

**Figure 2:**
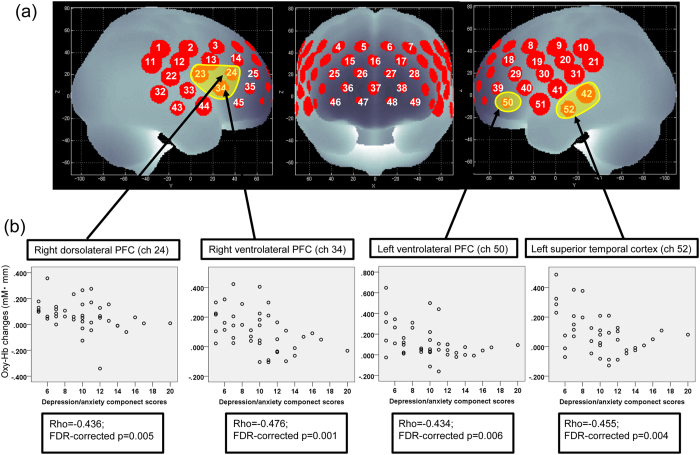
Cortical distribution of the areas of significant correlation between mean oxygenated haemoglobin (oxy-Hb) changes and depression/anxiety component scores. (**a**) Brain area in yellow corresponds to the near-infrared spectroscopy (NIRS) channels in which mean oxy-Hb changes show significant correlation with depression/anxiety component scores (Spearman’s correlation coefficient; false discovery rate (FDR)-corrected *p* < 0.05). The locations of NIRS channels were probabilistically estimated and anatomically labelled in the standard Montreal Neurological Institute brain space in accordance with Tsuzuki *et al*.^57^. (**b**) Scatter diagrams depicting the relationship between depression/anxiety component scores and mean oxy-Hb changes in channels 24 (right dorsolateral prefrontal cortex (PFC)), 34 (right ventrolateral PFC), 50 (left ventrolateral PFC), and 52 (left superior temporal cortical).

The task procedure was similar to that of Takizawa *et al*.^9^. Hb changes were measured during the VFT (letter version). Each subject sat in a comfortable chair during the NIRS measurements and was instructed to minimize movement of the head, strong biting, and eye blinking to avoid artefacts. The 160-s block-design VFT contained three different periods: a 30-s pre-task period, 60-s task period, and 70-s post-task period (Fig. 2). For the pre- and post-task baseline periods, participants were instructed to repeat consecutively the five Japanese vowels (a, i, u, e, and o) aloud. Hb change was calculated as the difference in concentration between the syllable-repeating condition in the pre-task baseline period and the verbal fluency condition in the task period. The subjects were required to repeat syllables during the baseline periods in the VFT, instead of remaining silent as in other studies. This procedure enables one to differentiate between the utterance process and the word-generating process^17^.The contrast between the verbal fluency condition and vocalization condition was used to increase the specificity for verbal fluency of the readout from the NIRS. During the task period, participants were instructed to generate as many Japanese words beginning with a designated syllable as possible. The three sets of initial syllables (A: /to/, /se/, /o/; B: /a/, /ki/, /ha/; C: /na/, /i/, /ta/) were presented in a counterbalanced order among the participants, and each syllable was changed every 20 s during the 60-s task. The total number of correct words generated during the VFT was adopted as the measure of task performance.

### NIRS methodology

A 52-channel NIRS (ETG-4000; Hitachi Medical Co.) machine was used to measure changes in Hb concentration. The NIRS probe comprised 3 × 11 arrays with 17 emitters and 16 detectors. The NIRS probe machine was placed on the fronto-temporal region of each participant, with the midcolumn of the probe located over the Fpz and the lowest probes located along the T3-Fp1-Fpz-Fp2-T4 line, in accordance with the international 10–20 System used in electroencephalography. The distance between pairs of source-detector probes was set at 3 cm, and each measurement area between the pairs of probes was defined as a “channel” (ch). The machine measures activities along the light path at a depth of 2–3 cm below the scalp, which corresponds well with the surface of the cerebral cortex^53^,^54^. According to the LONI Probabilistic Brain Atlas (LPBA40)^55^, NIRS channels can record bilateral functional hemodynamics within the frontal, temporal, and parietal cortices. Similar to our previous study^9^,^56^, we anatomically labelled NIRS channels only after the LPBA region of highest probability was determined. The spatial information from each channel was estimated using the functions from the Functional Brain Science Laboratory at the Jichi Medical University in Japan (http://www.jichi.ac.jp/brainlab/virtual_reg.html)^57^.

NIRS measures change in both oxy-Hb and deoxy-Hb at two wavelengths (695 and 830 nm) of infrared light, based on the modified Beer–Lambert law^58^. In this NIRS system, these Hb values include a differential path length factor (DPF); therefore, Hb concentrations from baseline to activation periods were recorded. The relative changes in Hb concentration were indicated as millimoles per millimetre.

The sampling frequency was 10 Hz. To examine VFT-related activities, data for Hb changes were analysed according to the “integral mode”: the pre-task baseline was determined as the mean across 10-s just before the task period, while the post-task baseline was determined as the mean across 5-s, 50-s after the task period. Assuming that task-related activities returns to a pre-task baseline level at a constant interval, linear fitting was performed using the Hb data obtained between the pre-task and post-task baselines. The same procedure has been often used in NIRS studies^9^,^17^,^18^ (Fig. 2). A moving-average method, involving the use of a 5-s window, was applied to remove any short-term motion artefacts. In addition, noise related to body-movement artefacts (no signal, high frequency, and low frequency) were rejected using the algorithm published by Takizawa *et al*.^9^,^18^.

The mean changes in oxy-Hb (as opposed to those in deoxy-Hb) were measured during the VFT as an index of cortical activity because oxy-Hb better reflects this activity and shows stronger correlations with blood oxygenation level-dependent signals as measured by fMRI^15^,^59^,^60^, although the analyses of the mean changes in deoxy-Hb are also presented.

### Statistical Analysis

Statistical analyses were performed using SPSS 19.0 software (Tokyo, Japan).

The mean Hb changes of the pre-task baseline period and that of the task period were compared using paired two-tailed *t*-tests for each channel to confirm the activities area during the VFT.

Because the Shapiro–Wilk test revealed that the original three-factor (positive, negative, and general psychopathology) and five-factor components of the PANSS are not normally distributed, Spearman’s correlation coefficients (r) were calculated to explore the relationships between mean Hb changes during the VFT and the original three-factor and five-factor components of the PANSS. In addition, to elucidate the independent contributions of the depressive symptom components of the PANSS to mean Hb changes in those channels that showed significant correlations, we performed stepwise multiple regression analyses. In these analyses, the mean Hb changes were the dependent variables, and other potential confounding variables were controlled, such as age, gender (dummy parameterized, male = 1, female = 0), premorbid IQ, task performance on the VFT, Global Assessment of Functioning (GAF) score, duration of illness, and daily dosage of antipsychotic drugs used in the analyses of schizophrenia, with a probability of F for a conservative entry and removal criteria of 0.05 and 0.2, respectively. For significant findings, effect sizes were indicated using the standardized regression coefficient (β). Statistical significance was set at *p* < 0.05, and multiple comparisons among the 52 channels were corrected using the false discovery rate (FDR) method^61^.

## Additional Information

**How to cite this article**: Pu, S. *et al*. Associations between depressive symptoms and fronto-temporal activities during a verbal fluency task in patients with schizophrenia. *Sci. Rep.* **6**, 30685; doi: 10.1038/srep30685 (2016).

## Figures and Tables

**Table 1:** Patient demographics and clinical characteristics.

	N = 41 (mean ± SD)
Age, years	33.6 ± 11.2
Gender, women/men	23/18
Handedness	96.4 ± 12.1
Education, years	13.7 ± 2.2
Estimated premorbid IQ	100.1 ± 11.3
Number of words generated	12.2 ± 3.7
Age at onset, years	22.5 ± 8.8
Duration of illness, years	11.1 ± 9.1
GAF	52.0 ± 9.4
PANSS	
Total	62.4 ± 15.4
Positive	13.2 ± 4.3
Negative	17.7 ± 5.0
General psychopathology	31.6 ± 8.7
Five-factor model of the PANSS	
Positive component	7.9 ± 2.9
Negative component	15.9 ± 5.2
Excitement component	6.7 ± 2.3
Depression/anxiety component	9.9 ± 3.6
Cognitive comonent	7.6 ± 2.2
Antipsychotic medication, mg/day (in chlorpromazine equivalents)	526.1 ± 363.1
Antidepressant medication	N (%) 3 (7.3), 2 on Duloxetine, 1 Sertraline

Abbreviations: IQ, Intelligence Quotient; GAF, Global assessment of functioning; PANSS, Positive and Negative Symptom Scale.

**Table 2:** Summary of stepwise multiple regression analysis in channels showing significantly correlated with depression/anxiety component scores of the PANSS.

No. of channels^a^	R^2^	Adjusted R^2^	Independent Variables		Other Factors
			Depression/anxiety component scores of the PANSS		
			*β*	*P*	
Right ventrolateral PFC					
Ch23	0.323	0.282	−0.375	0.016	Premorbid IQ: *β* = 0.344, *P* = 0.026
Ch34	0.196	0.176	−0.443	0.004	
Right dorsolateral PFC					
Ch24	0.115	0.093	−0.340	0.030	
Left ventrolateral PFC					
Ch50	0.155	0.133	−0.393	0.011	
Left temporal					
Ch42	0.178	0.156	−0.422	0.007	
Ch52	0.299	0.260	−0.517	0.001	Gender: *β* = −0.326, *P* = 0.029

Note: PANSS, Positive and Negative Syndrome Scale; No., number; Ch, channels; PFC., prefrontal cortex; IQ, intelligence quotient.

^a^Depression/anxiety component scores of the PANSS, age, gender, premorbid IQ, task performance on the verbal fluency task, Global Assessment fo Functioning, duration of illness, and daily dosage of antipsychotic drugs were included in the multiple linear regression analysis.
